# Serum Uric Acid Is Associated with Erectile Dysfunction: A Population-Based Cross-Sectional Study in Chinese Men

**DOI:** 10.1038/s41598-017-02392-x

**Published:** 2017-05-18

**Authors:** Fengbin Gao, Boren Jiang, Zhen Cang, Ningjian Wang, Bing Han, Qin Li, Yi Chen, Yingchao Chen, Fangzhen Xia, Hualing Zhai, Chi Chen, Meng Lu, Ying Meng, Yingli Lu, Zhoujun Shen

**Affiliations:** 1 Department of Urology, Ruijin Hospital, Shanghai Jiaotong University School of Medicine, Shanghai, 200025 China; 2 Institute and Department of Endocrinology and Metabolism, Shanghai Ninth People’s Hospital, Shanghai JiaoTong University School of Medicine, Shanghai, 200011 China

## Abstract

The role that serum uric acid (UA) plays in the pathophysiological development of erectile dysfunction (ED) is controversial. We aimed to screen the factors related with ED, and to examine the association between serum UA and ED. Our data were derived from a cross-sectional Survey on Prevalence in East China for Metabolic Diseases and Risk Factors study in 2014–2015. Questionnaire of International Index of Erectile Dysfunction-5 was used for assessment of ED. Data were collected in three general communities respectively. A total of 1365 men were enrolled with an overall mean age 55.5 ± 10.8 years (range: 20–83 years). The prevalence of ED was 62.4% (51.4% standardized) in the population. Males with ED were older, and more prone to have a higher follicle-stimulating hormone, luteinizing hormone, sex hormone-binding globulin, glycated hemoglobin, fasting plasma glucose levels and lower free androgen index (FAI), UA levels, and more likely to have diabetes and elevated blood pressure compared with those without ED. Age and UA were independent influencing factors for ED. Besides, UA was positively correlated with FAI after adjustment for age. In conclusion, our study demonstrated the protective role that UA might play in development of ED.

## Introduction

Erectile dysfunction (ED), a kind of male sexual dysfunction, is characterized by the inability to achieve or maintain erections of the penis potent for satisfaction during sexual activity^1^. Even though ED is not a fatal condition, it interferes with daily routine, social interactions and quality of life of the patient. ED is a common clinical problem, especially in aging men^2^, and the number of patients suffered from ED will be up to 322 million by 2025 worldwide^3^. Given the etiology of ED is multifactorial, pathophysiology of ED is influenced by several physical systems^4^.

Uric acid (UA), the end product of dietary and endogenous purine metabolism, is currently confirmed to modulate the physiological functions of various physical systems. Several epidemiological cohort studies have demonstrated that the level of serum UA is associated with many clinical events, such as metabolic syndrome, hypertension, and diabetes^5^. So far, several experimental and clinical studies on the fields of the relationship between UA and ED have given clues on the potential causative role of UA in ED occurrence^6, 7^. However, most of the above deductions were based on the researches which were restricted to patients with particular diseases, such as hypertension^8^ and coronary artery disease^9^, hence, the generalization of these studies are limited. To confirm the connection between ED and UA, large-scale and population-based studies which provide convincing evidence are needed.

A large population-based study, referred to as Survey on Prevalence in East China for Metabolic Disease and Risk Factors (SPECT-China), was launched in 2015–2016. Based on the analysis of the data from the survey, we attempted to explore the independent influential factors for ED and the linkage of ED with UA.

## Results

### Characteristics of the study population

Table 1 summarized the general demographic and laboratory characteristics of the participants who were divided into two groups according to whether the investigators were suffering from ED or not. A total of 1365 men met the inclusion criteria for this study with an overall mean age 55.5 ± 10.8 years (range: 20–83 years). Compared with the men without ED, the men in ED group were older and had significantly lower serum UA levels, higher glycated hemoglobin (HbA1c), fasting plasma glucose (FPG) and systolic blood pressure (SBP) levels. Moreover, the ED group possessed a higher prevalence of diabetes. As to the sex-related hormones, the men with ED had significantly lower free androgen index (FAI) and higher sex hormone-binding globulin (SHBG), follicle-stimulating hormone (FSH) and luteinizing hormone (LH) levels. Differences in total testosterone (TT), body mass index (BMI) and diastolic blood pressure (DBP) levels were not statistically significant between the two groups.

**Table 1:** General characteristics of the participants.

N	Total	Men without ED	Men with ED
	1365	513	852
Age (year)	55.5 ± 10.8	51.6 ± 10.7	57.9 ± 10.3**
Sex-related hormones			
TT (nmol/L)	17.2 ± 6.3	16.9 ± 6.1	17.4 ± 6.3
FSH (IU/L)	8.6 ± 6.5	7.5 ± 5.8	9.3 ± 6.8**
LH (IU/L)	6.3 ± 3.3	5.9 ± 2.9	6.6 ± 3.5**
SHBG (nmol/L)	45.8 ± 22.4	42.3 ± 21.3	48.0 ± 22.8**
FAI (%)	42.5 ± 14.5	45.8 ± 16.0	40.7 ± 13.1*
Metabolic factors			
HbA1c (%)	6.0 ± 1.1	5.8 ± 0.9	6.1 ± 1.1**
FPG (mmol/L)	5.7 ± 1.6	5.5 ± 1.2	5.8 ± 1.7**
UA (μmol/L)	343.6 ± 77.1	349.9 ± 76.2	339.8 ± 77.4*
BMI (kg/m^2^)	25.5 ± 3.1	25.6 ± 3.0	25.4 ± 3.1
Diabetes (%)	19.4	14.6	22.3**
SBP (mmHg)	136.8 ± 20.4	133.5 ± 19.3	138.7 ± 20.9**
DBP (mmHg)	84.7 ± 13.1	84.3 ± 12.9	85.0 ± 13.2

The data are summarized as the mean ± standard deviation for continuous variables, or as number with proportion for categorical variables. The Mann–Whitney U test was used for non-normally distributed continuous variables, and the Pearson χ2 test was used for dichotomous variables

*P < 0.05, significantly lower than men without ED group.

**P < 0.05, significantly higher than men without ED group.

Abbreviations: ED, erectile dysfunction; TT, total testosterone; FSH, follicle-stimulating hormone; LH, luteinizing hormone; SHBG, sex hormone binding globin; FAI, free androgen index; HbA1c, glycated hemoglobin; FPG, fasting plasma glucose; UA, uric acid; BMI, body mass index; SBP, systolic blood pressure; DBP, diastolic blood pressure.

**Figure 1:**
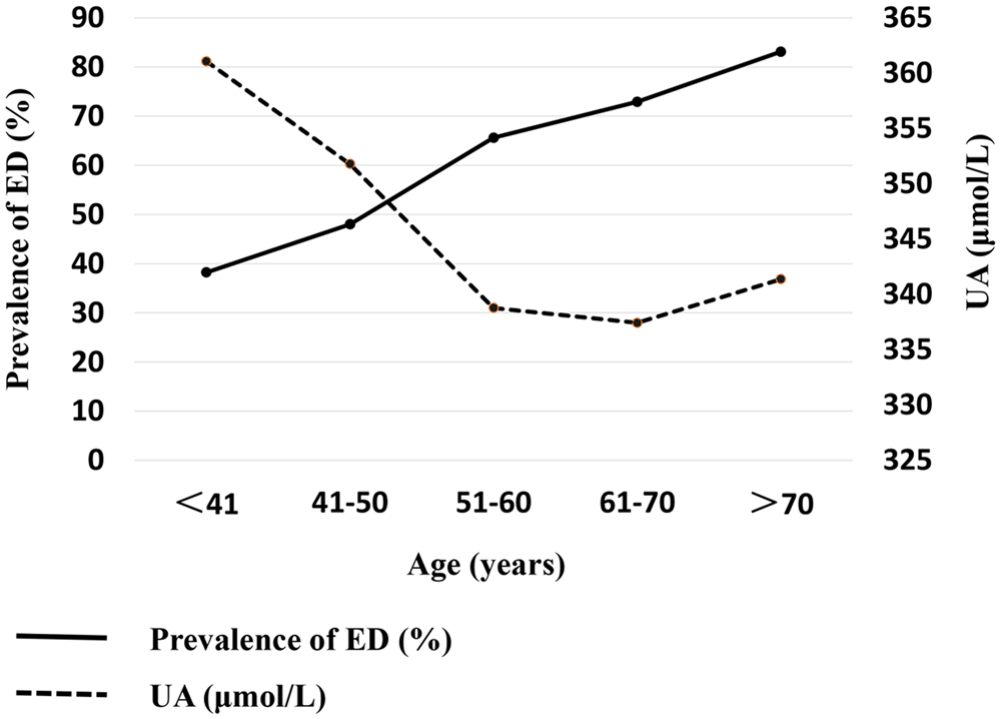
Changes in prevalence of ED and the concentration of UA by age groups. The horizontal axis, the left vertical axis, and the right vertical axis represent age groups, the prevalence of ED, and the serum UA concentration respectively.

Figure 1 illustrated the prevalence of ED and the concentration of serum UA across age groups. Overall, the prevalence of ED was 62.4% (51.4% standardized by age, referred to the 6^th^ national population census data, www.stats.gov.cn). The percentiles of ED were initially low (38.2%) at ages less than 40 years and then increased progressively with increasing age. We noticed that the prevalence of ED increased an average 10 percent per decade approximately. On the contrary, the serum UA concentration showed a declining trend with the increase of age generally.

Table 2 showed the characteristics of the study population according to serum UA quartiles. The quartile ranges of UA were ≤289 μmol/L (UA1), 290–341 μmol/L (UA2), 342–396 μmol/L (UA3) and ≥397 μmol/L (UA4). With the increasing concentration of UA, the prevalence of both ED and diabetes had significantly descendent tendency. Meanwhile, HbA1c and age had the same trend with diabetes, however, conversely, DBP presented an escalating trend. Besides, in terms of sex-related hormones, compared with men in the lowest quartile, the men in the highest quartile had significantly lower TT, FSH, LH and SHBG levels but higher FAI level.

**Table 2:** Characteristics of the participants by quartiles of UA.

N	UA1	UA2	UA3	UA4
	342	343	341	339
Age (year)	56.5 ± 10.1	55.8 ± 10.9	55.3 ± 10.8	54.5 ± 11.5*
Sex-related hormones				
TT (nmol/L)	18.9 ± 6.7	17.8 ± 6.0*	16.8 ± 5.8*	15.2 ± 5.8*
FSH (IU/L)	8.9 ± 7.3	9.1 ± 6.0	8.4 ± 6.2	8.0 ± 6.2*
LH (IU/L)	6.5 ± 3.4	6.5 ± 3.1	6.2 ± 3.4	6.1 ± 3.2
SHBG (nmol/L)	54.0 ± 26.1	48.3 ± 21.9*	43.2 ± 18.6*	37.8 ± 19.1*
FAI (%)	39.3 ± 12.3	40.9 ± 12.9	43.3 ± 14.5**	47.8 ± 17.3**
Metabolic factors				
HbA1c (%)	6.2 ± 1.4	5.9 ± 1.0*	5.9 ± 0.8*	5.9 ± 0.8*
FPG (mmol/L)	6.0 ± 2.1	5.6 ± 1.5	5.5 ± 1.2	5.6 ± 1.2
BMI (kg/m^2^)	24.4 ± 3.0	25.1 ± 2.7**	25.8 ± 2.9**	26.6 ± 3.2**
Diabetes (%)	26.9	17.7*	14.9*	17.9*
SBP (mmHg)	135.1 ± 20.8	137.2 ± 20.9	136.7 ± 20.5	137.9 ± 19.6
DBP (mmHg)	82.3 ± 13.0	85.1 ± 13.0**	85.2 ± 13.7**	86.3 ± 12.5**
ED (%)	67.2	62.6	59.8*	59.7*

The quartile ranges of UA were ≤ 289 μmol/L (UA1), 290–341 μmol/L (UA2), 342–396 μmol/L (UA3) and ≥397 μmol/L (UA4).

The data were summarized as the mean ± standard deviation for continuous variables, or as number with proportion for categorical variables. The Mann–Whitney U test was used for non-normally distributed continuous variables, and the Pearson χ2 test was used for dichotomous variables.

*P < 0.05, significantly lower than UA1.

**P < 0.05, significantly higher than UA1.

Abbreviations: UA, uric acid; TT, total testosterone; FSH, follicle-stimulating hormone; LH, luteinizing hormone; SHBG, sex hormone binding globin; FAI, free androgen index; HbA1c, glycated hemoglobin; FPG, fasting plasma glucose; BMI, body mass index; SBP, systolic blood pressure; DBP, diastolic blood pressure; ED, erectile dysfunction.

### UA was an independent influential factor for ED

To explore the independent risk or protective factor for erectile function, binary logistic regression analysis was performed. Univariate logistic regression analysis showed that age [Odds ratio (OR), 95% confidence intervals (CI)] (1.057,1.046–1.069); FSH(1.054,1.031–1.077); LH (1.076,1.037–1.117); SHBG (1.012,1.007–1.017); HbA1c (1.265,1.125–1.421); FPG (1.125,1.040–1.217); systolic pressure (1.013,1.007–1.017) were significantly associated with increased odds of ED, on the contrary, both FAI (0.974,0.965–0.983) and UA (0.998,0.997–1.000) had protective effect on erectile function (Table 3). TT, BMI were not significantly associated with ED.

**Table 3:** Influential factors for ED

Factors	Univariate analysis			Multivariate analysis		
	OR	95%CI	*P*	OR	95%CI	*P*
Age (year)	1.057	1.046, 1.069	<0.001	1.049	1.031, 1.067	<0.001
FSH (IU/L)	1.054	1.031, 1.077	<0.001	1.019	0.978, 1.062	0.373
LH (IU/L)	1.076	1.037, 1.117	<0.001	0.997	0.931, 1.067	0.933
FAI (%)	0.974	0.965, 0.983	<0.001	0.997	0.986, 1.008	0.554
HbA1c (%)	1.265	1.125, 1.421	<0.001	1.108	0.961, 1.276	0.157
UA (μmol/L)	0.998	0.997, 1.000	0.019	0.996	0.992, 1.000	0.020
BMI (kg/m^2^)	0.979	0.945, 1.014	0.240	—	—	—
SBP (mmHg)	1.013	1.007, 1.017	<0.001	1.006	0.999, 1.013	0.115

Binary logistic regression was used to examine the influential factors for ED.

Abbreviations: FSH, follicle-stimulating hormone; LH, luteinizing hormone; SHBG, sex hormone binding globin; FAI, free androgen index; HbA1c, glycated hemoglobin; UA, uric acid; BMI, body mass index; SBP, systolic blood pressure; ED, erectile dysfunction; OR, odds ratio; CI, confidence internal.

Based on the multivariate logistic regression analysis, the adjusted OR of age to ED was 1.048 (1.030–1.067), and the OR of UA to ED was 0.998 (0.996–1.000). The results confirmed that age was an independent risk factor for ED, and UA was an independent protective factor (Table 3).

Moreover, according to the univariate logistic regression results in Table 3, HbA1c and FPG, two well-known indexes related to diabetes, were both closely associated with deterioration of erectile function. Consistent with the above data, men with diabetes were with an increased prevalence of ED compared with men without diabetes (72.3% vs 60.1%, P < 0.001, Supplementary Table S1). Based on the median of UA concentration, we divided the men with diabetes into low UA group (UA ≤ 320 μmol/L) and high UA group (UA > 320 μmol/L), and analyzed the difference of ED prevalence in the two groups to further investigate the effect of UA on erectile function in patients with diabetes. As shown in Supplementary Table S1, the prevalence of ED in low UA group was slightly higher than that in high UA group, though there was no significant difference (P = 0.585).

### Association between UA and sex-related hormones

Table 3 revealed that FAI, LH and FSH were closely related with ED (P < 0.001), however, multivariate logistic regression analysis showed they were not independent influential factors. Hence, we attempted to explore the association between UA and sex-related hormones. As previously stated according to the data in Table 2, the men with higher UA concentration had significantly lower TT, FSH, LH and SHBG levels but higher FAI level. To further clarify the relationship between UA and sex-related hormones, linear regression models were utilized. As shown in Table 4, the higher levels of UA were associated with higher FAI (P < 0.001), and lower TT (P < 0.001), FSH (P = 0.008), LH (P = 0.007) and SHBG (P < 0.001) in the unadjusted models. Because UA and sex hormones were all significantly related to age (data not shown), hence, all the following linear regression analysis between UA and sex-related hormones were adjusted for age. After adjustment, TT, SHBG and FAI were still significantly associated with UA (all P < 0.001), however, the association of UA with FSH and LH weakened further to the extent that they were no longer significant.

**Table 4:** Association of UA with sex-related hormones.

	UA*			UA**		
	B	95%CI	P	B	95%CI	P
TT (nmol/L)	−2.669	−3.334, −2.063	<0.001	−2.591	−3.235, −1.947	<0.001
FSH (IU/L)	−0.858	−1.486, −0.229	0.008	−0.501	−1.196, 0.195	0.158
LH (IU/L)	−1.703	−2.938, −0.468	0.007	−1.069	−2.406, 0.268	0.117
SHBG (nmol/L)	−0.957	−1.132, −0.781	<0.001	−1.029	−1.224, −0.833	<0.001
FAI (%)	1.118	0.802, 1.435	<0.001	0.993	0.621, 1.364	<0.001

Linear regression analysis was used.

*was unadjusted; **was adjusted by age.

Abbreviations: UA, uric acid; TT, total testosterone; FSH, follicle-stimulating hormone; LH, luteinizing hormone; SHBG, sex hormone binding globin; FAI, free androgen index; B, regression coefficient; CI, confidence internal.

## Discussion

ED is a global problem that influences a great proportion of males. In our demographic study, the intact data of in total 1365 males was collected for systematic analysis. The prevalence of ED in our study (62.4%) is similar to several population-based studies^10–12^. Given the geographical and cultural variation, the statistical data of the prevalence of ED range from 16.1% to 80.8%^11, 13–15^ with considerable differences. Hence, ED is a worldwide detriment to male health, and the influential factors need to be well clarified for diagnose and treatment. Our primary finding is that elevated serum UA level is an independent protective factor for ED. As far as we know, it is the first time to elaborate the relationship between UA and ED through a large population-based cross-sectional study.

Frankly speaking, one contentious area is whether UA plays a protective role for ED or not. Various reports holding the opposite view have described that UA was a potential risk factor for the presence of ED. In a case-control study, compared with the normal group, the serum UA levels of males with ED elevated significantly (P < 0.001)^7^. Additionally, the data of two studies focused on the males with hypertension^8^ and coronary artery disease^9^ respectively, revealed the detrimental role that UA played in the process of ED. The conclusions of the above studies are hard to be persuasive because several great limitations exist, for example, the results are not universal due to the small sample size and the research methods such as case-control are liable to cause selection bias. Their results may be applicable for specific population groups with particular disease, and the viewpoint that UA is a risk factor for ED lacks evidences derived from large population-based cross-sectional studies for supporting. By contrast, our demonstration of the association between UA and ED based on demographic analysis is more universal and representative. Although the mechanism is not clearly illustrated, we attempt to elucidate the linkage between UA and ED from the following points.

Initially, depression, a kind of psychogenic problems, is considered to be directly correlated with ED^16^. Meanwhile, recent studies have found low UA level was associated with depression^17, 18^. In a case-control study, lower UA level was a characteristic of depression compared with the healthy group, in addition, the UA level raised to 312.28 μmol/L from the original 271.97 μmol/L after a five-week treatment with antidepressants (P = 0.043). Similarly, a multivariate analysis conducted by Gu *et al*. revealed that the declining serum UA promoted the development of depression^19^. Given the linkage between UA and depression, it provides an explanation on the protective role that UA plays in maintaining the erection of penis by alleviating depression.

As a vascular event, penile erection requires an intact endothelium to occur, and endothelial dysfunction is a manifestation of ED^20^. Oxidative stress impairs the vascular endothelium by enhancing production of reactive oxygen species (ROS)^21^, consequently contributes to the pathogenesis of ED^22^. Hyperglycemia induces abnormalities of vascular endothelium by promoting release of ROS^23, 24^. Consistently, in our study, the men suffered from diabetes were with a high prevalence of ED. Besides, numerous studies have provided evidence that UA is a powerful scavenger of ROS^25^. For example, a randomized, double-blind, placebo-controlled study has reported that administration of UA raises circulating antioxidant defense and accelerates the restoration of endothelium-dependent vasodilation which represents the improvement of endothelial function^26^. Furthermore, a crossover study conducted by Waring has validated that substantial reduction of serum UA concentrations is invalid for restoring endothelial function in patients with diabetes^27^. Therefore, UA may be beneficial for erectile function through its powerful antioxidative effect on endothelium protection to some extent. And the viewpoint may give a reasonable explanation for the results in our study that the prevalence of ED decreases with the increasing concentration of UA. Moverover, with regard to the patients with diabetes, our study demonstrates that ED is more prevalent in the lower UA group than in higher UA group, which is consistent with Waring’s study.

Androgens are indispensable in maintaining erectile response, and the underlying molecular mechanisms have been clarified clearly during the past decade^28^. In our study, we also confirmed that ED prevalence was inversely correlated with FAI, but not TT. An explanation is that the level of androgenicity may not be exactly reflected by TT due to the inactive part which binds with circulating SHBG^29^. Subsequently, we delineated the positive correlation between UA and FAI, which is in accordance with previous researches^30, 31^. In Kurahashi’s study, after the androgen replacement therapy, the UA concentration increased and hyperuricemia was more prevalent in the high-androgen-dose group^31^. Trials on laboratory animals have demonstrated that androgens promote UA reabsorption in the kidney^30^. To sum up, androgens may prevent the deterioration of erectile function by raising the serum UA concentration.

Based on our results of multivariate logistic regression analysis, serum UA is an independent protective factor for ED. The OR of UA is 0.998, which is very close to 1, indicating that the effect of UA on ED is modest. And this could partly explain why a very small numerical difference in UA concentrations exists between ED and non-ED groups (339.8 ± 77.4 vs 349.9 ± 76.2 μmol/L). In addition, we conclude that serum UA levels increase with age which supporting the major effect of age on UA. The mean age of men with ED group is distinctly higher than that of men without ED group. Hence, if age is adjusted, UA concentration in ED group would be lower than 339.8 μmol/L, and the difference of serum UA between the two groups would be larger.

Our study had several strengths. First, quality of the study was controlled strictly, and the same trained research group completed all the anthropometric measurements and questionnaires, meanwhile, all the biochemical and hormonal items were examined in one laboratory certified by the College of American Pathologists. Second, our results are more universal because the study was performed with large sample size. However, some important limitations must be recognized. First, due to the cross-sectional nature of the study, we cannot identify the causality but only the association between serum UA level and ED. Second, this study recruited mainly Han Chinese men and the results cannot apply to other ethnic groups. Third, the sample in our study was a relatively older population with the mean age 55.5 years, and the sample size in each age group had a certain difference. Although International Index of Erectile Dysfunction-5 (IIEF-5) is widely recognized for assessment of ED in both clinical and epidemiologic studies, dividing sharply whether suffering from ED or not according to the IIEF-5 score below 22 is not appropriate. Lastly, ED, which is closely related with self-esteem, is a highly private topic for males. Hence, the possibility exists that parts of the participants didn’t fill in the IIEF-5 questionnaire honestly, and the uncontrolled factor may cause bias.

In conclusion, based on this population-based cross-sectional study, we estimated the prevalence of ED in Chinese males. Age, SHBG, FAI, FSH, LH, HbA1c, FPG, diabetes, systolic pressure and UA had strong association with ED. Among them, UA was an independent protective factor of ED. In addition, UA had a notable positive correlation with FAI.

## Methods

### Study population

The survey, SPECT-China, is a population-based cross-sectional study on the prevalence and influential factors of metabolic disease. The registration number is ChiCTR-ECS-14005052 (www.chictr.org.cn)^32, 33^. To obtain more persuasive and generalized data, a stratified cluster sampling method was adopted. Our investigation covered both rural and urban areas, meanwhile, different economic development of area was considered. This investigation was conducted in Shanghai, Anhui Province and Jiangsu Province. Followings were the inclusion criteria: male adults aged 18 years and older; living in the current area for 6 months or longer; and Chinese citizens. Participants were excluded if they unwilling to participate; received diagnosis of serious psychiatric diseases; used drugs that affected sexual function and experienced no sex life for 6 months prior to the date of the investigation.

Altogether 2178 participants took part in the study. The respondents who refused completing questionnaire (n = 414), had no sex life within the last six months (n = 287) and incomplete laboratory or questionnaire data (n = 112) were excluded. Finally, a total of 1365 men were enrolled in the study. Prior to the data collection of this research, all the participants signed written informed consent. The investigation was approved by the Ethics Committee of Shanghai Ninth People’s Hospital, Shanghai Jiao Tong University School of Medicine. In accordance with the Declaration of Helsinki, the research was carried out.

### Measurements

A standard procedure was executed by the same trained staff group to complete questionnaire. The questionnaire was split into two parts: one included information on demographic characteristics, medical history and lifestyle, the other is the IIEF-5 questionnaire, which is used to estimate the severity of ED^34^. Subsequently, the collection of anthropometric data, such as body weight, height and blood pressure, was used standard methods as described previously^35^. To protect participants’ privacy and to ensure data authenticity and reliability, IIEF-5 questionnaires were completed in a separate room. To obtain the accurate biochemical indexes and hormone levels of blood samples, all the participants were asked to fast overnight at least 8 h before the study. The blood samples for the plasma glucose test were collected into anticoagulant vacuum tubes and centrifuged on the spot within 1 hour after collection. Blood samples were stored at −20 °C after being collected and shipped by air on dry ice. A central laboratory, certified by the College of American Pathologists, was responsible for the measurement of blood samples indexes. HbA1c was assessed by HPLC (MQ-2000PT, Medconn, Shanghai, China). FPG was measured by a Beckman CoulterAU680. Sex-related hormones including TT, LH and FSH were measured via chemiluminescence using the Immulite 2000 platform (Siemens, Munich, Germany). The missing values of TT under the minimal detectable limit were replaced by the mean values that were 0.35 nmol/L between 0 and the minimal detectable limit. SHBG levels were detected via electrochemiluminescence using a Cobas E601 analyzer (Roche, Switzerland). BMI was calculated as weight in kilograms divided by height in meters squared. FAI, which may reflect increased androgenicity properly^36^, is the ratio of total circulating testosterone to SHBG.

### Definition of variables and outcomes

In regard of the assessment of ED, participants with the IIEF-5 questionnaire score below 21 were classified into the ED group, and the rest with score 22–25 were considered with normal erectile function^34^. Diabetes was defined as fasting plasma glucose ≥7.0 mmol/L, or HbA1c ≥6.5% based on the American Diabetes Association 2014 criteria.

### Statistical analysis

The analysis of the collected data was performed with IBM SPSS Statistics, version 22 (IBM Corporation, Armonk, N.Y., USA). The mean values and a number with proportion were adopted to summarize the general characteristics for continuous variables and categorical variables respectively. Kolmogorov–Smirnov analysis was used for normality testing. For abnormal distribution data, Mann–Whitney U test was used. A logistic regression analysis was used to evaluate the risk factors for ED. All considered variables were introduced in the univariate and multivariate model, meanwhile, OR and 95% CI were presented. Linear regression analysis was used for examining the association between UA and sex-related hormones. The conventional P < 0.05 was used to assess statistical significance.

## Electronic supplementary material

Supplementary Table S1
