# Supplementary material for: Serum Uric Acid Is Associated with Erectile Dysfunction: A Population-Based Cross-Sectional Study in Chinese Men
Source: Sci Rep. 2017 May 18;7:2087. doi: 10.1038/s41598-017-02392-x (PMC5437090; doi:10.1038/s41598-017-02392-x)
Supplement: Supplementary file 1 — Supplementary Table S1 [file 41598_2017_2392_MOESM1_ESM.pdf]

Serum Uric Acid Is Associated with Erectile Dysfunction: A Population-Based Cross-Sectional Study in Chinese Men

Fengbin Gao[1,+], Boren Jiang[2,+], Zhen Cang[2], Ningjian Wang[2], Bing Han[2], Qin Li[2], Yi Chen[2], Yingchao Chen[2], Fangzhen Xia[2], Hualing Zhai[2], Chi Chen[2], Meng Lu[2], Ying Meng[2], Yingli Lu[2]*, and Zhoujun Shen[1]*.

	Men without diabetes	Men with diabetes		
		Low UA	High UA	Total
N	1101	132	132	264
UA(μmol/L)	346.7±75.8	264.4±37.8	396.2±54.9 ##	330.3±81.0 *
Age(year)	54.9±11.2	58.8±8.4	57.6±9.5	58.2±8.9 **
HbA1c(%)	5.6±0.5	7.8±1.7	7.1±1.0 #	7.5±1.4 **
FPG(mmol/L)	5.2±0.5	8.4±2.7	7.4±1.9 #	7.9±2.4 **
BMI(kg/m^2)	25.3±3.1	25.6±2.9	26.6±3.1 ##	26.1±3.0 **
ED(%)	60.1	74.2	70.4	72.3 **

Supplementary Table S1. Correlation between UA and ED prevalence in patients with diabetes

The data are summarized as the mean ± standard deviation for continuous variables, or as number with proportion for categorical variables. The Mann–Whitney U test was used for non-normally distributed continuous variables, and the Pearson $\chi 2$ test was used for dichotomous variables

*P < 0.05, significantly lower than men without diabetes.

**P < 0.05, significantly higher than men without diabetes.

#P < 0.05, significantly lower than low UA group.

##P < 0.05, significantly higher than low UA group.

Abbreviations: UA, uric acid; ED, erectile dysfunction; HbA1c, glycated hemoglobin; FPG, fasting plasma glucose; BMI, body mass index.
